# Label-Free Monitoring of Endometrial Cancer Progression Using Multiphoton Microscopy

**DOI:** 10.1007/s10439-024-03574-1

**Published:** 2024-07-03

**Authors:** Xuzhen Wu, Yanqing Kong, Yu Yi, Shuoyu Xu, Jianhua Chen, Jianxin Chen, Ping Jin

**Affiliations:** 1 Department of Gynecology, Shenzhen Maternity and Child Healthcare Hospital, Shandong University, Shenzhen, 518028 China; 2 Department of Pathology, Shenzhen Maternity and Child Healthcare Hospital, Shenzhen, 518028 China; 3 Key Laboratory of OptoElectronic Science and Technology for Medicine of Ministry of Education, Fujian Provincial Key Laboratory of Photonics Technology, Fujian Normal University, Fuzhou, 350117 China; 4 Department of General Surgery, Nanfang Hospital, Southern Medical University, Guangzhou, 510515 China; 5 College of Life Science, Fujian Normal University, Fuzhou, 350117 China

**Keywords:** Endometrial cancer, Multiphoton microscopy, Interstitial collagen, Tumor progression

## Abstract

Endometrial cancer is the most common gynecological cancer in the developed world. However, the accuracy of current diagnostic methods is still unsatisfactory and time-consuming. Here, we presented an alternate approach to monitoring the progression of endometrial cancer via multiphoton microscopy imaging and analysis of collagen, which is often overlooked in current endometrial cancer diagnosis protocols but can offer a crucial signature in cancer biology. Multiphoton microscopy (MPM) based on the second-harmonic generation and two-photon excited fluorescence was introduced to visualize the microenvironment of endometrium in normal, hyperplasia without atypia, atypical hyperplasia, and endometrial cancer specimens. Furthermore, automatic image analysis based on the MPM image processing algorithm was used to quantify the differences in the collagen morphological features among them. MPM enables the visualization of the morphological details and alterations of the glands in the development process of endometrial cancer, including irregular changes in the structure of the gland, increased ratio of the gland to the interstitium, and atypical changes in the glandular epithelial cells. Moreover, the destructed basement membrane caused by gland proliferation and fusion is clearly shown in SHG images, which is a key feature for identifying endometrial cancer progression. Quantitative analysis reveals that the formation of endometrial cancer is accompanied by an increase in collagen fiber length and width, a progressive linearization and loosening of interstitial collagen, and a more random arrangement of interstitial collagen. Observation and quantitative analysis of interstitial collagen provide invaluable information in monitoring the progression of endometrial cancer. Label-free multiphoton imaging reported here has the potential to become an in situ histological tool for effective and accurate early diagnosis and detection of malignant lesions in endometrial cancer.

## Introduction

Endometrial cancer is the most common malignancy of the female genital tract in the United States with an estimated 65,950 new cases and 12,550 deaths in 2022 [1]. In 2014, the World Health Organization modified the classification of endometrial hyperplasia to include only two categories: (1) hyperplasia without atypia and (2) hyperplasia with atypia, i.e., atypical hyperplasia or endometrial intraepithelial neoplasia (EIN). Studies have shown that about 1–3% of hyperplasia without atypia cases may progress to highly differentiated endometrial cancer, while about 30% or even 40% of atypical hyperplasia /EIN cases may coexist with cancer [2]. Accurate monitoring of the progression of endometrial cancer will facilitate early detection of lesions, early intervention, and determination of the appropriate treatment scope to avoid over- or under-treatment.

Histological evaluation of biopsy or curetting specimens with hematoxylin and eosin (H&E) staining is the gold standard for diagnosing endometrial hyperplasia and endometrial cancer. Their pathology is mainly characterized by a clonal proliferation of endometrial glands lined or not lined by atypical cells, with a predominance of glands over the interstitium [3]. However, this diagnostic method is limited by many objective reasons: (1) sampling error, loss of specimen and destruction of its integrity; (2) the inevitable subjectivity arising from the dependence on the experience of pathologists; and (3) disturbances from the same pathological characteristics present in lesions such as hormonal irregularities, regeneration, and metaplastic changes [4]. These uncertainties make it challenging to reach definitive conclusions. Therefore, it is necessary to explore a new method to solve these problems and monitor the progression of endometrial cancer.

As one of the main components of the extracellular matrix (ECM), collagen, which is often overlooked in routine pathological diagnosis of endometrial hyperplasia and endometrial cancer, has been confirmed to be crucial in tumor genesis and development [5, 6]. Provenzano et al. observed three tumor-associated collagen signatures, enabling the identification of pre-palpable breast tumors [7]. Li et al. found that collagen content in the gastric tissues increased as the gastric cancer progressed [8]. The enhanced stiffness of ECM is known as a result of a stabilized accumulation of collagen by increased protein concentration, increased cross-linking, or parallel reorientation [9]. This loss of tissue homeostasis is associated with the development of cancer [10]. However, Arifler et al. found different results in oral biopsies, in which the neoplastic stroma, instead of hardening, was degraded to produce collagen fibers with looser and more disordered in appearance because the degraded collagen fibers were shorter, more detached from each other, and had irregularly aggregation tendency than normal stroma [11]. Similarly, the normal cross-linking of collagen provides the elasticity, structural integrity, and tensile properties required for normal uterine function. Abnormal cross-linking of collagen causes lesions such as preterm birth attributed to decreased cervical cross-link density and uterine fibroids attributed to increased cross-link density [12]. Not coincidentally, Jussila et al. observed an increased collagen synthesis in the stroma of well-differentiated endometrial adenocarcinomas, while decreased deposits of collagens in moderately differentiated tumors [13]. These results suggest the complex changes of collagen in different cancer species. Therefore, collagen monitoring is advantageous as part of a holistic strategy to help detect, diagnose, and identify lesions earlier. Disappointingly, the traditional pathological method cannot quantitatively or qualitatively assess collagen content and distribution. Furthermore, collagen status in endometrial cancer remains unclear, just as the correlation between changes in collagen and the development of endometrial cancer has yet to be carefully studied until this work.

Multiphoton microscopy (MPM) based on two-photon excited fluorescence (TPEF) and second-harmonic generation (SHG) has become increasingly popular in the studies of the structure of biological tissues. It can capture TPEF signals generated by endogenous fluorescent substances in cells, such as NADH and FAD. These fluorescent substances vary with the metabolic state of the cells and cause changes in TPEF signals when cells become cancerous [14, 15]. In addition, collagen has a non-centrosymmetric architecture or hyperpolarizability that makes it an SHG-active molecule [16, 17]. The harmonic signals generated by collagen fibers are captured by MPM for imaging, which provides a possibility to quantitatively monitor the morphological changes of collagen fibers during the disease progression process [18]. By combining TPEF and SHG signals, label-free MPM provides details of cells and collagen fibers in the ECM and can assist in monitoring the development of cancer [19, 20]. Unfortunately, the monitoring of endometrial cancer progression based on MPM has not been reported, to the best of our knowledge.

In this study, we obtained and analyzed 689 MPM images of 39 endometrial specimens, including normal endometrium, hyperplasia without atypia, atypical hyperplasia, and endometrial cancer (endometrioid adenocarcinoma), to demonstrate the capability of MPM to identify the morphological details and alterations of the glands and collagen morphological features during the formation of endometrial cancer. These data provide a valuable basis for the establishment of new methods for monitoring endometrial cancer.

## Methods

### Patients

In this study, endometrial pathological sections were collected from 17 patients attending the gynecology department of Shenzhen Maternity and Child Healthcare Hospital, including ten normal, nine hyperplasia without atypia, ten atypical hyperplasia, and ten endometrial cancer (endometrioid adenocarcinoma) cases. None of these patients were on hormone therapy. The specimens were obtained from the tissue of curettage or total hysterectomy. The study was approved by the institutional review board of Shenzhen Maternity and Child Healthcare Hospital.

### Multiphoton Imaging System

A commercial laser scanning microscope (LSM 880, Zeiss, Germany) equipped with a mode-locked femtosecond Ti:sapphire laser (Chameleon Ultra, Coherent, USA) was used to obtain multiphoton images. In this work, two independent channels were selected to collect two-photon excitation fluorescence (TPEF) and second-harmonic generation (SHG) signals, respectively. One channel covered a wavelength range of 395–415 nm to collect the SHG signal (color-coded green), while the other channel covered a wavelength range of 430–695 nm to display the TPEF signal (color-coded magenta) [21]. MPM images were obtained by scanning unstained histological section using a Plan-Apochromat×20 objective (*NA* = 0.8, Zeiss, Germany) and the images were further amplified by ZEN 2.3 SP1 software. The resolution of MPM images was 0.554 μm/pixel.

### Quantification of Collagen Morphological Features

**Fig. 1:**
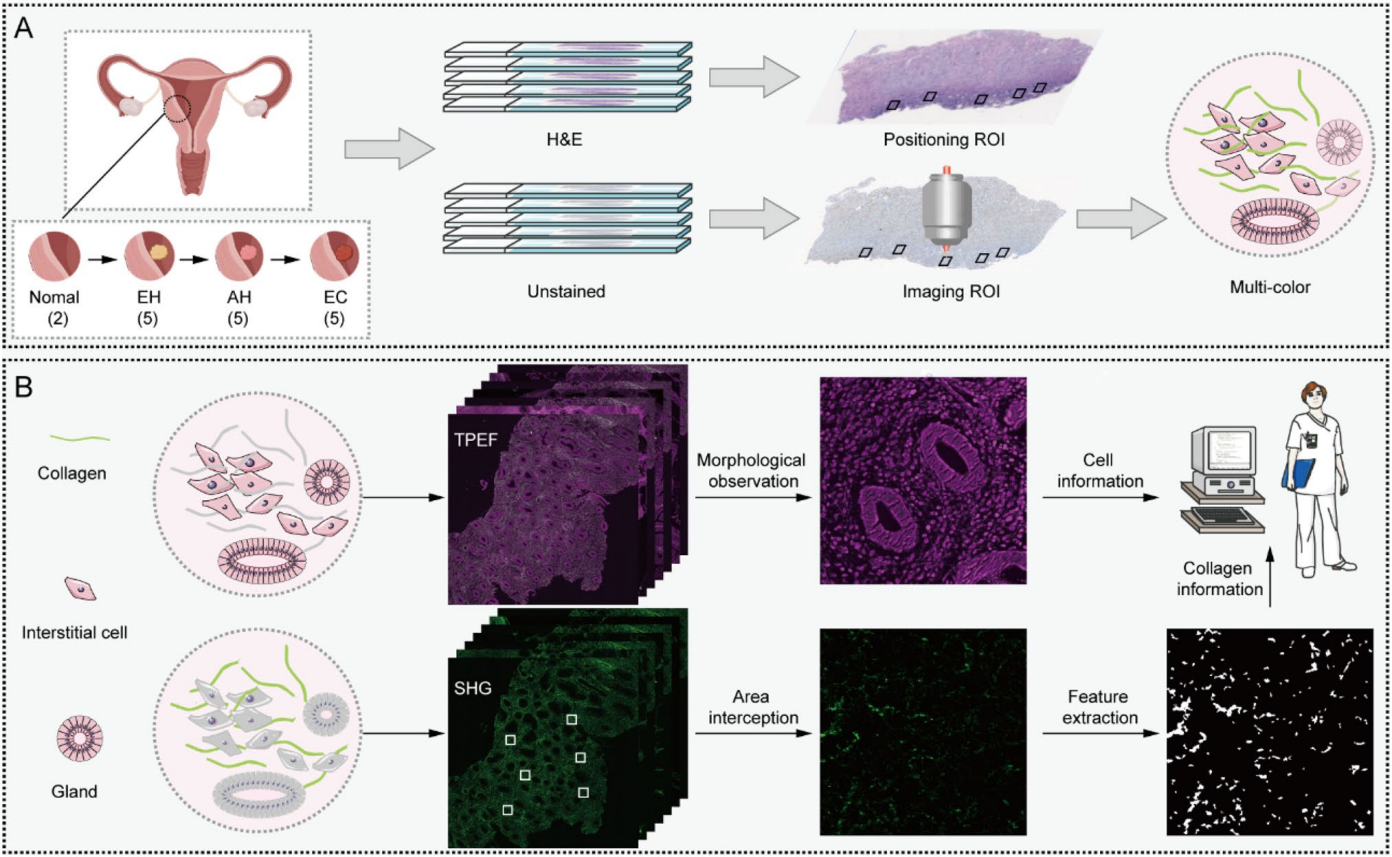
Schematic diagram and the ability of MPM to monitor endometrial cancer progression. **A** The flow of sample processing to distinguish the multi-color-coded histopathological features. **B** MPM can provide cytological information based on the principle of endogenous fluorescence imaging and microscopic morphological information of collagen based on the principle of non-centrosymmetric architecture imaging

Specimens were sectioned into two slices with 5 μm thickness. One slice was to locate the regions of interest (ROI) with H&E staining and the other was dewaxed to acquire MPM imaging without staining. Specifically, 3–5 non-overlapping regions of interest (ROI) with a size of about 2.2 × 2.2 mm^2^ were marked in H&E images. On another slice without staining, the corresponding positions of ROI were used to obtain the MPM image (Fig. 1A). Subsequently, in each ROI, 6 interstitial regions (150 × 150 μm^2^ for each region) near the endometrial glands were randomly intercepted to extract and quantify eight corresponding collagen morphological features by MATLAB 2016b (https://github.com/qldqq1984/CollagenFeature), including collagen proportionate area, collagen fiber number, collagen fiber length, collagen fiber width, collagen fiber straightness, collagen fiber cross-link density, collagen fiber cross-link space, and collagen fiber orientation (Fig. 1B) as previously described [22–24]. The extraction process consisted of the following steps. First, the SHG image was binarized into collagen fibers pixels and background pixels by using the Gaussian mixture model method [25]. Second, the fiber network extraction algorithm was used to identify and track each collagen fiber in the binary image [22]. Subsequently, the skeleton of each extracted collagen fiber was identified and represented by a list of ordered vertices [23]. If any vertex in the list belongs to more than one fiber, the software recognizes it as a cross-link point. The vertex list was used to calculate fiber density, length, width, straightness, cross-linking density, and cross-linking space. Finally, Fast Fourier Transformation was used to measure and quantify the degree of organization and symmetry of collagen fibers in the SHG image. Specifically, in the binarized images, the ratio of short axe to long axe was used to estimate the collagen orientation index, calculated by the index = [1 − (short/long)]. The value was between 0 and 1. A value close to 0 indicates the collagen fibers are isotropic, and a value close to 1 indicates the collagen fibers are more aligned in a particular direction [26–28].

### Statistical Analysis

Statistical analyses were done in SPSS (version 23.0). Quantitative data were expressed as mean ± standard deviation (SD). One-way analysis of variance (ANOVA) was used to compare more than two groups of data. All statistical tests were two-sided, and *P* values of less than 0.05 were deemed significant.

## Results

MPM images of endometrium in normal, hyperplasia without atypia**,** atypical hyperplasia, and endometrial cancer tissues.

**Fig. 2:**
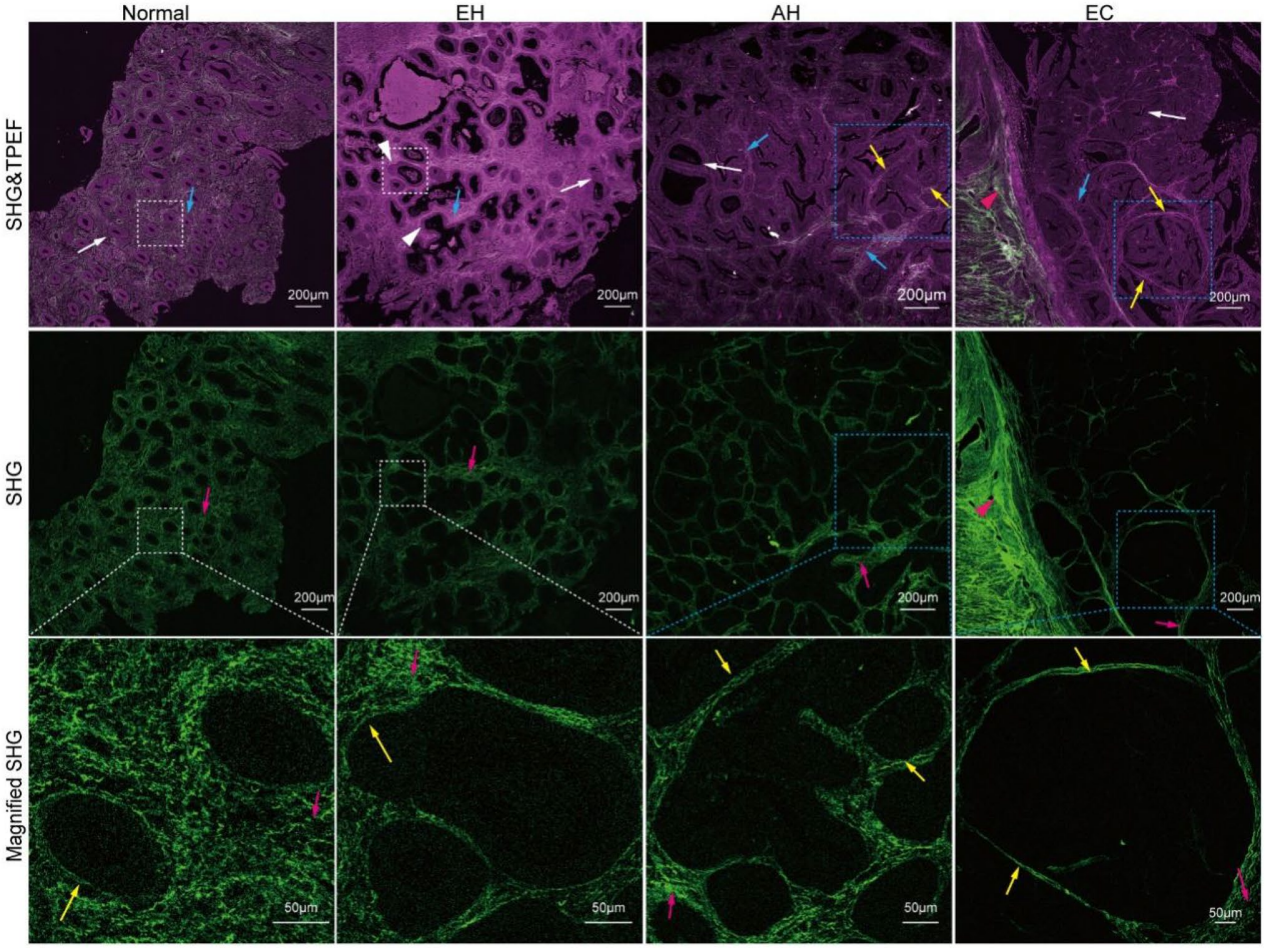
Representative MPM images (top panel), SHG images (middle panel), and zoomed-in SHG images (bottom panel) from normal, hyperplasia without atypia, atypical hyperplasia, and endometrial cancer tissues. *EH* hyperplasia without atypia, *AH* atypical hyperplasia, *EC* endometrial cancer. White arrow: gland, Blue arrow: interstitial cells, Pink arrow: collagen fibers, Yellow arrow: basement membrane, White arrowhead: budding or branching gland, Pink arrowhead: myometrium, White dashed box: individual glands, Blue dashed box: clustered glands

**Fig. 3:**
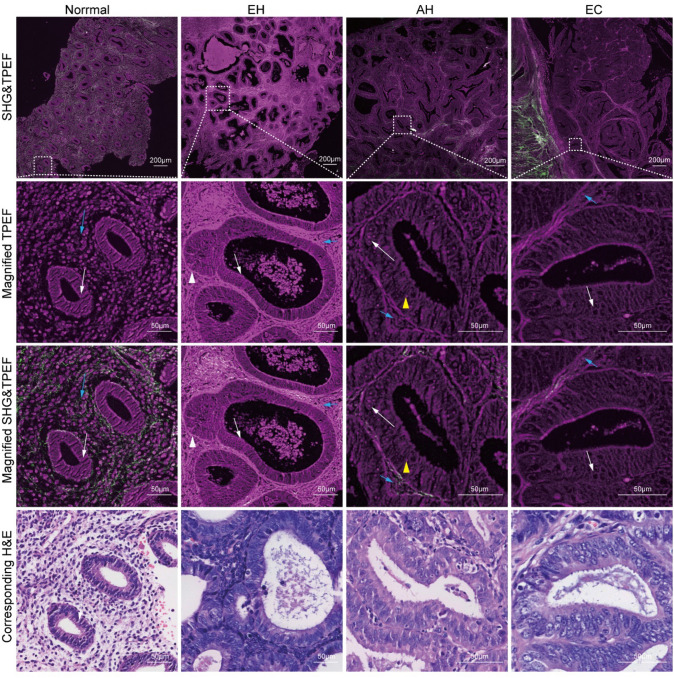
Magnified gland in TPEF images, SHG & TPEF images, and the corresponding H&E images of normal, hyperplasia without atypia, atypical hyperplasia, and endometrial cancer tissues. *EH* hyperplasia without atypia, *AH* atypical hyperplasia, *EC* endometrial cancer. White arrow: epithelial cell, Blue arrow: interstitial cells, White arrowhead: budding gland, Yellow arrowhead: nucleoli

In the process of endometrial cancer formation, pathological features of endometrial tissue are characterized by endometrial glands undergoing disordered proliferation and an increased ratio of glands to interstitium. To identify the architectural feature of endometrial glands in the microenvironment, we imaged endometrial glands in structurally normal, hyperplasia without atypia, atypical hyperplasia, and endometrial cancer tissues, respectively (Fig. 2). The glands in normal endometrial tissue are characterized by straight, small and relatively uniform glands, which are oval or round (white arrow in Fig. 2). Outer margins of the glands are smooth, flat, and surrounded by an intact basement membrane (yellow arrow in Fig. 2). The ratio of glands to interstitium (blue arrow in Fig. 2) is approximately 1:1. Collagen fibers, a major connective tissue in the endometrium, are finely reticulated (pink arrow in Fig. 2). Compared with normal tissue, the glands in hyperplasia without atypia are significantly more numerous, with a slight decrease in the proportion of interstitium. In hyperplasia without atypia endometrial tissue, the glands are dilated, and varied in size and morphology, with some glands budding or branching (white arrowhead in Figs. 2 and 3). Although the basement membrane around these glands is still intact, it is deformed at the location where the glands are budded or branched. With the development of hyperplasia without atypia, the enlarged glands are clustered in some areas of atypical hyperplasia (blue dashed box in Fig. 2), but the individual gland is still wrapped by the basement membrane (yellow arrow in Fig. 2). The interstitium and interstitial collagen fibers are rarely found between glands in atypical hyperplasia, but are still present. Different from normal, hyperplasia without atypia and atypical hyperplasia tissues, the number of glands in endometrial cancer increases markedly. In the endometrial cancer tissue, most glands proliferate outward and break through the basement membrane to fuse, which leads to the destruction of the basement membrane in SHG images and the formation of gland groups (blue dashed box) in MPM images (Fig. 2). Some of them have invaded the myometrium (pink arrowhead in Fig. 2). In addition, in endometrial cancer, gland fusion results in the reduction or loss of interstitium between glands.

Additionally, with the development of the disease, a series of detailed structural changes in glandular epithelial cells and quantitative changes in interstitial cells are observed in TPEF images. The epithelial cells of glands in normal endometrial tissue are monolayered and columnar, closely arranged with distinct cell polarity (white arrow in Fig. 3). The spindle-shaped interstitial cells are scattered in the interstitium (blue arrow in Fig. 3). In hyperplasia without atypia tissue, the epithelial cells are monolayer columnar or pseudostratified structure. Although the cells are similar in shape to normal endometrial epithelial cells, they are more loosely arranged than normal endometrial epithelial cells. Different from normal endometrium, the interstitial cells of hyperplasia without atypia are densely arranged. Budding or branching of hyperplasia without atypia glands (white arrowhead in Fig. 3) leads to the compression of interstitial space and dense arrangement of interstitial cells. With the progression of hyperplasia without atypia, the epithelial cells in atypical hyperplasia tissue exhibit heteromorphism with rounded nuclei, distinct nucleoli (yellow arrowhead in Fig. 3), and loss of cell polarity. The interstitium and its internal substances, such as interstitial cells, are diminished, but still present. The interstitial cells in endometrial cancer tissue are stratified or pseudostratified, significantly larger than normal epithelial cells, and have a distinct heteromorphism with enlarged, hollow nuclei. The interstitial cells, are very scarce and disappear in the gland fusion groups.

MPM image data confirm the morphological details and alterations of the glands during the formation of endometrial cancer, including the irregular changes in the structure of the gland, increased ratio of the gland to the interstitium, the atypical changes in the glandular epithelial cells and the destruction of basement membrane caused by gland proliferation and fusion.

Quantitative analysis of collagen morphology in normal, hyperplasia without atypia, atypical hyperplasia, and endometrial cancer tissues.

**Fig. 4:**
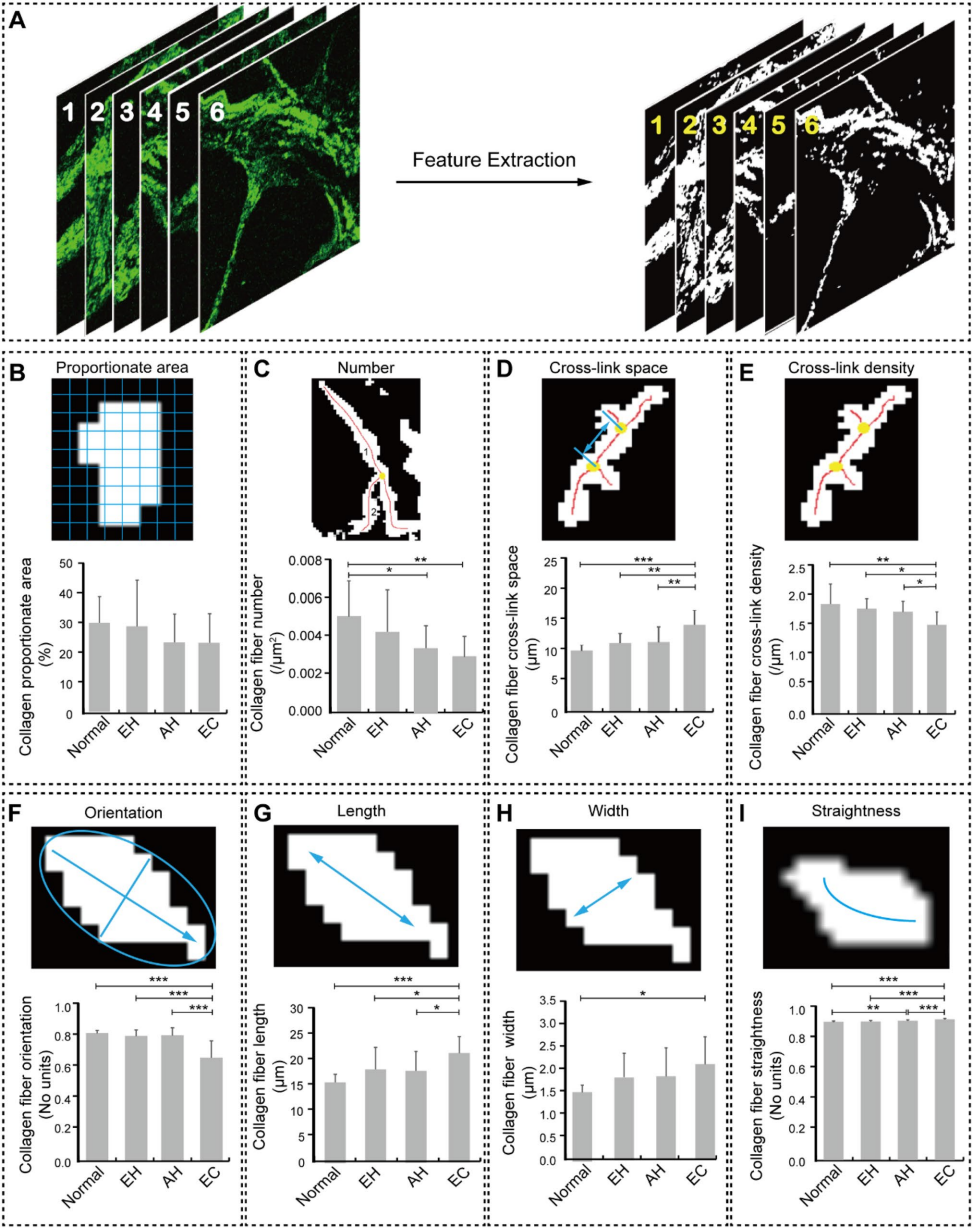
Schematic of extracting collagen morphological microscopic features. **A** The captured SHG image was divided into collagen fibers and a background. **B** Schematic of quantitative extraction of collagen proportionate area and its quantitative result in normal, hyperplasia without atypia, atypical hyperplasia, and endometrial cancer. **C** Schematic of quantitative extraction of collagen fiber number and its quantitative result in normal, hyperplasia without atypia, atypical hyperplasia, and endometrial cancer.** D** Schematic of quantitative extraction of collagen fiber cross-link space and its quantitative result in normal, hyperplasia without atypia, atypical hyperplasia, and endometrial cancer. **E** Schematic of quantitative extraction of collagen fiber cross-link density and its quantitative result in normal, hyperplasia without atypia, atypical hyperplasia, and endometrial cancer. **F** Schematic of quantitative extraction of collagen fiber orientation and its quantitative result in normal, hyperplasia without atypia, atypical hyperplasia, and endometrial cancer. **G** Schematic of quantitative extraction of collagen fiber length and its quantitative result in normal, hyperplasia without atypia, atypical hyperplasia, and endometrial cancer. **H** Schematic of quantitative extraction of collagen fiber width and its quantitative result in normal, hyperplasia without atypia, atypical hyperplasia, and endometrial cancer. **I** Schematic of quantitative extraction of collagen fiber straightness and its quantitative result in normal, hyperplasia without atypia, atypical hyperplasia, and endometrial cancer. The quantitative results were presented as mean ± SD. ^*^ *P < *0.05, ^**^ P < 0.01, ^***^ P < 0.001. *EH* hyperplasia without atypia, *AH* atypical hyperplasia, *EC* endometrial cancer

To more accurately observe changes in collagen morphology, a customized program was written for the automatic analysis of collagen morphological features in MPM images. In this program, the SHG images were binarized (Fig. 4A) and eight morphological features of interstitial collagen were extracted for quantitative analysis (Fig. 4B–I) by the software MATLAB. The program was applied to position the collagen and automatically calculate the pixels belonging to collagen in the segmented image. The collagen proportionate area and the collagen fiber number are two features to measure the content of collagen in the interstitium. Collagen proportionate area is defined as the percentage of pixels in the segmented image. The quantitative analysis results show that in the interstitium near endometrial glands, there is no significant difference in the collagen proportionate area among normal (29.9%), hyperplasia without atypia (28.8%), atypical hyperplasia (23.3%), and endometrial cancer (23.2%, Fig. 4B) tissues. The collagen fiber number, which is defined as the number of collagen fibers extracted per square micron in the segmented image, is 0.0051 ± 0.0019/μm^2^ in normal and 0.0043 ± 0.0022/μm^2^ in hyperplasia without atypia, and there is no significant difference between them. However, the collagen number in atypical hyperplasia (0.0034 ± 0.0012/μm^2^) and endometrial cancer (0.0030 ± 0.0011/μm^2^) are significantly less than that of normal endometrium, suggesting that the decrease of collagen number in the interstitium near endometrial glands is may be closely associated with the formation of atypical hyperplasia and endometrial cancer (Fig. 4C).

To further characterize the extent of interstitial changes during the formation of endometrial cancer, collagen fiber cross-link space, collagen fiber cross-link density, and collagen fiber orientation were analyzed. Fig. 4D shows the segmentation results of collagen fiber cross-link space, which is defined as the average distance between adjacent cross-link points for each fiber in the segmented image. Although there is no significant difference in collagen fiber cross-link space among normal endometrium (10.023 ± 0.881 μm), hyperplasia without atypia (11.187 ± 1.587 μm), and atypical hyperplasia (11.439 ± 2.460 μm), they are significantly smaller than that of endometrial cancer (14.127 ± 2.286 μm), which indicates that collagen fiber tends to be loose during the formation of endometrial cancer. As a feature corresponding to collagen fiber cross-link space, collagen fiber cross-link density is defined as the ratio of the total number of cross-link points to the sum of lengths of all collagen fibers in the segmented image. The result of quantitative analysis demonstrates that there is no significant difference in collagen fiber cross-link density among normal endometrial tissue (1.850/μm), hyperplasia without atypia (1.766/μm), and atypical hyperplasia (1.721/μm). In contrast, the collagen fiber cross-link density in endometrial cancer (1.505/μm) is significantly lower than that in normal endometrium (*P* < 0.01), hyperplasia without atypia (*P* < 0.05), and atypical hyperplasia (*P* < 0.05). The downward trend of collagen fiber cross-link density in endometrial cancer still suggests that the formation of endometrial cancer is accompanied by the loosening of collagen fibers (Fig. 4E). The quantitative analysis of collagen fiber orientation, which is defined as the principal direction determined according to the angular orientation distribution in the Fourier transformed image, also shows the similar downward trend. Specifically, there is no significant difference in collagen fiber orientation among normal endometrial tissue (0.814), hyperplasia without atypia (0.798), and atypical hyperplasia (0.801), while the collagen fiber orientation in endometrial cancer (0.658) is significantly lower than that in normal (*P* < 0.001), hyperplasia without atypia (*P* < 0.001), and atypical hyperplasia (*P* < 0.001), suggesting that the formation of endometrial cancer is accompanied by random arrangement changes of collagen fibers (Fig. 4F).

To more accurately explore reasons why the collagen proportionate area does not change significantly but the collagen fiber number decreases significantly in endometrial cancer formation, collagen fiber length, collagen fiber width, and collagen fiber straightness were quantified. Figure 4G illustrates the segmentation result of collagen fiber length, which is defined as the sum of the distances between adjacent vertices. The mean ± SD of collagen fiber length in normal endometrium, hyperplasia without atypia, and atypical hyperplasia are 15.403 ± 1.526 μm, 17.907 ± 4.384 μm, and 17.601 ± 3.879 μm, respectively, and there was no significant difference between them. Notably, the collagen fiber length in endometrial cancer (21.179 ± 3.249 μm) is significantly higher than that in normal endometrium (*P* < 0.001), hyperplasia without atypia (*P* < 0.05), and atypical hyperplasia (*P* < 0.05), indicating a trend of increased collagen fiber length during the formation of endometrial cancer. Collagen fiber width is defined as the average distance between a vertex and its nearest background pixel. The result of the quantitative analysis shows that the collagen fiber width is 1.501 ± 0.151 μm, 1.821 ± 0.527 μm, and 1.834 ± 0.638 μm in normal endometrium, hyperplasia without atypia, and atypical hyperplasia, respectively, and there was no significant difference between them. Nonetheless, the collagen fiber width in normal endometrium is significantly smaller than that of endometrial cancer (2.116 ± 0.595 μm) (Fig. 4H), indicating that the formation of endometrial cancer is accompanied by an increased width of collagen fibers. Collagen fiber straightness, which is defined as the distance between the first and last vertices divided by their length, has a similar trend to collagen fiber length during the formation of endometrial cancer. The mean and SD of collagen fiber straightness in normal endometrium are 0.897 ± 0.003, which is not statistically different from that in the hyperplasia without atypia (0.901 ± 0.004). Although the collagen fiber straightness in atypical hyperplasia (0.905 ± 0.008) is not significantly different from that in hyperplasia without atypia, it is significantly higher than that in the normal endometrium (*P* < 0.01). Similar to the change of collagen fiber length in endometrial cancer, the collagen fiber straightness in endometrial cancer (0.914 ± 0.05) is significantly higher than that in normal endometrium (*P* < 0.001), hyperplasia without atypia (*P* < 0.001), and atypical hyperplasia (*P* < 0.001) (Fig. 4I). This trend of increasing collagen fiber straightness shows a linear trend of collagen fibers.

In summary, quantitative analysis of collagen morphological features reveals that the formation of endometrial cancer is accompanied by increased collagen fiber length and width, progressively linearized and loosened interstitial collagen, and a more random arrangement of interstitial collagen.

## Discussion

The incidence of endometrial cancer shows a global increase [29]. Although most endometrial cancer cases occur after the age of 50, it is more common to diagnose the disease before delivery due to the delayed onset of reproductive age [30]. Since different types of hyperplasia have different risks of turning into endometrial cancer, effective disease monitoring and consequently the optimal treatment not only reduce the likelihood of endometrial cancer forming but also help to protect the patient’s fertility. However, due to the limitations of existing diagnostic procedures, as discussed earlier, the misdiagnosis rate is 10% [31]. Therefore, it is of clinical significance to develop a new and improved diagnostic technique.

By capturing the fluorescent compounds naturally present in endometrial tissue, such as NAHD, FAD, and collagen [32], MPM images reveal that the formation of endometrial cancer is accompanied by an incremental increase in the number of glands and a progressive disorder and irregularity of glands. With the proliferation and fusion of glands, the interstitium progressively diminishes and even completely disappears during the formation of endometrial cancer, which is consistent with the pathological changes shown in traditional H&E images. Notably, because the interstitium attached to the basement membrane of the gland is rich in collagen, SHG images clearly show the location of the basement membrane in situ, helping identify the glandular fusion and interstitial infiltration, which are key to distinguishing atypical hyperplasia and highly differentiated endometrial cancer. Therefore, MPM can clearly show the morphological changes of the glands in the microenvironment, and it is well suited for the visualization of the microenvironment during the formation of endometrial cancer. It not only has the same pathological recognition ability as traditional H&E staining technology but also offers the additional advantage of its visualization capability for the integrity of the gland basement membrane, which is often ignored by the traditional H&E method.

To more clearly monitor the microscopic changes of collagen fibers, eight morphological features of interstitial collagen in normal, hyperplasia without atypia, atypical hyperplasia, and endometrial cancer specimens were quantified. A progressive decrease in the number of collagen fibers during the formation of endometrial cancer is shown in SHG images. This result contrasts with findings in malignancies such as breast, stomach, and liver cancer reported in the literature. Previous studies have found that the microenvironment of deposited and increased collagen would be conducive to the development of cancer [33–35]. The development of endometrial cancer is typically characterized by increased glands and decreased interstitium, so collagen, the main component in the interstitium, inevitably decreases along with the increased occupation of glands in the interstitium. The reduction in collagen fiber number might reduce the binding force with the hyperplastic glands, which in turn would facilitate further proliferation of glands. However, the exact mechanism causing the reduction of the binding force needs to be further investigated. In addition, the progressive linearization of interstitial collagen in SHG images is consistent with the previous studies. Brian et al. [36] have found that the increased collagen fiber straightness promotes the development of ductal carcinoma in situ in the breast. Jia et al. have shown that linearized collagen promotes the invasion of breast cancer cells [37]. Similar to the above results, our observation also confirms the linearization of collagen with the formation of endometrial cancer, which might act as a migration channel for tumor cells to promote tumor development [38]. There is evidence from previous studies to suggest that the increased collagen fiber cross-link density would facilitate the development of hepatocellular carcinoma [34], and interstitial sclerosis caused by collagen deposition is associated with the increased aggressiveness of breast cancer [39]. It is worth noting that, different from the results of these reports, in our study, the formation of endometrial cancer is accompanied by decreased collagen fiber cross-link density, decreased collagen fiber orientation, and increased collagen fiber cross-link space. Nevertheless, our research is consistent with the results of some other types of cancers. For example, the development of pancreatic cancer is also accompanied by the reduction of collagen deposition [40, 41]. Therefore, the specific changes of interstitial collagen might depend on the tumor type. According to the literature, the activated cancer-associated fibroblasts (CAF) trigger the increase of matrix metalloproteinases (MMPs) activity and finally cut the interstitial collagen to reduce the diffusion resistance in the formation of endometrial cancer [6, 42–45]. Therefore, the loosened interstitial collagen suggests that the resistance to glandular proliferation is reduced, which could promote endometrial cancer carcinogenesis. Our study does not elucidate its specific mechanism, which is worth further research. In addition, compared with normal, hyperplasia without atypia, and atypical hyperplasia specimens, the increased width and decreased number of collagen fibers in endometrial cancer tissue explain the result that there is no significant difference in collagen proportionate area among normal, hyperplasia without atypia, atypical hyperplasia, and endometrial cancer specimens. In conclusion, the formation of endometrial cancer is accompanied by significant microscopic changes in collagen morphology, which gradually creates a microenvironment conducive to early endometrial cancer. These early microscopic changes of collagen fiber can be effectively monitored by the MPM-aided image automatic analysis method, which is impossible for the conventional H&E technology. The quantitative and qualitative analysis of interstitial collagen helps to timely monitor the occurrence of early endometrial cancer and initiate appropriate therapeutic measures to delay or even reverse the disease’s progress. To the best of our knowledge, this is one of the few (if not the first) studies to systematically investigate the collagen changes during the formation of endometrial cancer.

MPM imaging in endometrial cancer progression enables visualization of glandular cells and collagen fiber morphology, which has many advantages in the diagnosis of histopathology. First of all, MPM technology does not require markers and uses endogenous fluorescent substances for imaging, which avoids misdiagnosis caused by errors in staining. Secondly, our findings provide a theoretical basis for the clinical diagnosis of intravital MPM in future. The conventional methods for obtaining diagnostic specimens, namely endometrial biopsy and diagnostic scraping, are blind and often endanger the diagnosis due to insufficient samples obtained [46]. Although biopsy at hysteroscopy has improved the accuracy of sampling, it is still influenced by operator subjectivity. The lack of uniform standards for sampling and diagnosis also limits its popularity. Since MPM images can perform serial scans, virtual three-dimensional images of regions can be reconstructed by combining computer software, with a depth range of nearly 500 μm [47]. Combined with the nonlinear two-photon effect of near-infrared light, MPM could image deeper tissues with less photodamaging than fluorescence microscopy [48], which makes MPM very suitable for intravital diagnosis in early endometrial cancer. Encouragingly, some research on intravital MPM has made some achievements. For example, some studies have successfully used MPM to track the regression trajectory of MSCs and cellular responses in vivo [49, 50], and some research has used label-free multiphoton endoscopy to observe the deformation of regular crypt structures, the damage to the epithelium, and the formation of a collagen matrix in a mouse model of acute colitis [51]. Therefore, if hysteroscopy and MPM technology are combined, precise localization of the lesions and sufficient valuable specimens could be obtained in real time. These advantages of MPM would make it a useful tool to monitor the progression of endometrial cancer and may potentially replace the rapid frozen section for rapid intraoperative diagnosis, which could substantially reduce the time needed to guide the determination of the scope of surgery and even enable real-time decision-making on additional surgical procedures when the patient is still under anesthesia. Our study establishes a framework for the clinical application of MPM in the field of endometrial cancer.

## Conclusion

In summary, our results demonstrate that interstitial collagen changes both quantitatively and qualitatively during endometrial cancer progression and that this change can be used to help track the development of endometrial cancer. Label-free multiphoton imaging is feasible and effective in monitoring endometrial cancer progression by providing cellular and collagen morphological details. It not only has the advantages of H&E technology, but also has a better diagnostic performance in the microscopic diagnosis of collagen morphology. It has the potential to become an in situ histological tool for early diagnosis and detection of malignant lesions in endometrial carcinoma. With the progress of MPM-based hysteroscopic techniques and clinical applications, real-time in vivo assessment of tumor tissue using MPM may become the primary tool in endometrial cancer diagnosis.

## Data Availability

The datasets used and/or analyzed during the current study are available from the corresponding author upon reasonable request.

## Abbreviations

MPM: Multiphoton microscopy
TPEF: Two-photon excited fluorescence
SHG: Second-harmonic generation
MMP: Matrix metalloproteinase
H&E: Hematoxylin-eosin
